# Individualism and Egalitarianism Can Kill: How Cultural Values Predict Coronavirus Deaths Across the Globe

**DOI:** 10.3389/fpsyg.2021.620490

**Published:** 2021-06-22

**Authors:** C. Dominik Güss, Ma. Teresa Tuason

**Affiliations:** ^1^Department of Psychology, University of North Florida, Jacksonville, FL, United States; ^2^Clinical Mental Health Counseling, Department of Public Health, University of North Florida, Jacksonville, FL, United States

**Keywords:** cultural values, coronavirus deaths, individualism, collectivism, power distance, COVID-19

## Abstract

While in some countries, many people have died due to the coronavirus (COVID-19), in other countries, only a few have died. Based on the cultural values theory, our first hypothesis was that in countries that are predominantly individualistic, the number of deaths will be high, whereas in countries with predominantly collectivist values, the number of deaths will be low. Our second hypothesis was that countries with high power distance and hierarchy will have fewer deaths compared to countries with low power distance and egalitarianism. The hypotheses were tested by referring to two different value studies (Hofstede's study of 76 countries and Schwartz's study of 75 countries) while also controlling for GDP per capita, Gini index, population density, median age per country, and BMI per country. Of the five control variables GDP and BMI significantly predicted coronavirus deaths. Taking into account GDP, Gini index, population density, median age, and BMI, hierarchical regression analyses confirmed the first hypothesis on individualism and the second hypothesis on egalitarianism. Therefore, in the case of this current pandemic, group-oriented and collectivist values and low egalitarianism values lead to specific health-related behaviors that ultimately keep more people alive.

## Introduction

Almost every country in the world is battling the coronavirus (COVID-19) pandemic. As of October 1, 2020, over 33 million people worldwide (33,842,281) had contracted COVID-19, and over 1 million (1,010,634) people had died due to the virus (World Health Organization, 2020). The countries with the highest confirmed COVID-19 cases are the United States, Brazil, and India while those with the least number of cases are Fiji, Jamaica, and Namibia (World Health Organization, 2020). On the other hand, the countries with the highest death rates per 100,000 people are Belgium, Great Britain, and Spain, while those with the lowest death rates per 100,000 people are Taiwan, Thailand, and Jordan (Johns Hopkins University, 2020).

Although a country's wealth, healthcare resources, and technological advancements may be factors that lead to the successful handling of the crisis and reduced risk (Takian et al., 2020), they are not the only factors. The United States, for example, has a Gross Domestic Product (GDP) of $21M (World Bank), but it accounts for most deaths that have occurred due to COVID-19. As of July 18, 2020, there had been 137,674 deaths in the United States alone and in March 2021, there were over 500,000 deaths in the United States. On the other hand, many of the poorest countries, such as Fiji (with a GDP of $5,535), Jamaica (with a GDP of $16,458), and Namibia (with a GDP of $12,366), have handled the situation relatively well. Researchers have debated, and it has been found that social factors, such as inequality and poverty, lead to more increased exposure to the virus and increased risk of death (Elgar et al., 2020; Marmot and Allen, 2020; Patel et al., 2020). Thus, we assessed the relationship between a country's level of development—using the GDP per capita, the Inequality-adjusted Human Development Index (IHDI, which combines the three dimensions of health, education, and income in one score and adjusts for inequalities within countries), the Gini index (as a measure of income inequality), and healthcare-related expenses per capita—and the number of COVID-19 related deaths.

We argue that additional factors that exacerbate exposure to COVID-19 and increase the risk of death are certain socio-geographic features of a country, such as population density. One would argue that the more a country is densely populated, the higher the risks for exposure, spread, and death—a finding supported by Zhang and Schwartz (2020) when studied within the United States. However, some countries have been able to control the virus relatively well-despite population density, while some countries have not. For example, countries that are densely populated have sometimes managed to quickly enforce effective public health practices; one such example is Taiwan, which, due to its experiences with the SARS epidemic, has implemented the following policies: using facial masks, screening incoming travelers, using 4-h test kits, quarantining symptomatic patients, and employing rapid contact tracing and widespread testing (Chen, 2020; Lin et al., 2020; Wang C. J. et al., 2020). Thailand, another densely populated country, also managed to quickly enforce measures, such as having temperature checks in workplaces, imposing curfews along with the police guarding checkpoints, installing spraying stalls to disinfect people, and providing protective shields at street food stalls and for each table in schools (Beech, 2020). Thus, we wanted to assess whether population density relates to the spread of COVID-19.

Low infection rates in Thailand were also due to the partnership among various governmental and private sectors (Tantrakarnapa et al., 2020). Similarly, Jordan became one of the first countries to implement a severe lockdown (Alqutob et al., 2020); it closed airports, borders, and stores and was able to prioritize the needs of vulnerable groups such as the poor, children, elderly, and refugees. This was done by immediately distributing food to the poor, providing government assistance to 50% of their citizens, and banning companies from laying off their workers while still allowing companies to reduce employee wages and reduce public sector salaries (Arraf, 2020). As seen in these countries, as well as in a study of 84 countries, death rates have been impacted by societal governance factors such as confidence in state institutions, civic engagement, and implementation of certain policies and regulations of behaviors that are followed by their citizens (Elgar et al., 2020). Thus, in studying health behaviors surrounding COVID-19 and death risk, we are including government effectiveness, one of the six worldwide governance indicators, which specifically highlights the perceptions of the quality of public services independent from political pressures, the formulation of policies and their implementation, and the quality of infrastructures in serving the people (Kaufmann et al., 2010).

Equally important in health behaviors implemented by the government to decrease exposure to COVID-19 and to decrease the risk for mortality are individuals' protective health factors. As protective health factors, young age and low body mass index have been discussed (e.g., Tartof et al., 2020; Wang X. Q. et al., 2020). Conversely, obesity and older age are identified as factors that make people vulnerable to mortality among COVID-19 inpatients (Pettit et al., 2020). In addition, health behaviors, such as consistently wearing masks and face shields, staying 6 ft apart from people, staying away from crowds and places with poor ventilation, regular hand washing, etc. (Center for Disease Control Prevention, 2020), are related to fewer COVID-19 deaths (Conyon et al., 2020). These health behaviors, which mitigate the pandemic, are supported by extant social and behavioral research, including cultural values specified in individual and collective interests and the social and cultural context (Bavel et al., 2020). We thus argue that health behaviors are directly related to certain cultural values. Values are the guiding principles for people's thoughts and behaviors; they are desirable goals that motivate action (Kluckhohn and Strodtbeck, 1961; Schwartz, 1994; Kemmelmeier et al., 2002; Güss, 2011). Following cultural values theory, we argue that the cultural values of Individualism-Collectivism and Power Distance have a direct impact on people's behaviors that protect them from the virus exposure and, ultimately, on the number of COVID-19 related deaths.

Individualism refers to the dominant values in a society where people are loosely connected to each other and where the expectation is to care for oneself and one's immediate family only (Hofstede, 2001; Hofstede et al., 2010). Individualism prioritizes the pursuit of one's own ideas and satisfying one's needs for curiosity, freedom, independent enjoyment, and positive experience (Schwartz, 2020). Alternatively, collectivism in society exists when people are interconnected since birth, relationships are solid, and people watch out for each other (Hofstede, 2001; Hofstede et al., 2010). The focus is on how people regard collective identity, the meaning derived from these connections, and involvement in common goals and shared activities (Schwartz, 2020). Hypothesis 1: the extent to which a country is individualistic correlates to thinking of oneself only in this pandemic and behaving with lesser regard for the safety of others, such as not social distancing and not wearing face masks, thereby increasing exposure to the virus, which may increase the risk for coronavirus infections and deaths.

A second value dimension that possibly influences health behaviors related to coronavirus infections and deaths is Power Distance. According to Hofstede (Hofstede, 2001; Hofstede et al., 2010), Power Distance is the degree to which people in society assume and anticipate that power is unevenly distributed. In the case of high Power Distance, individuals consent to the hierarchy and to the uneven allocation of influence, authority, and wealth. Hypothesis 2: Our second hypothesis is that the extent to which people assume and accept unequal power distribution in a country and the extent to which powerful people make decisions for the less powerful in this pandemic, to mitigate the risk of exposure such as lockdowns, relates to fewer confirmed COVID-19 cases and deaths.

The scientific community has discussed whether it is legitimate to divide the one dimension into the two dimensions Individualism/Collectivism and Power Distance [see e.g., critique of Minkov et al. (2017) and Van de Vliert and Kong (2019)] as Hofstede has done since the two dimensions correlate highly with each other [*r* = −0.55, see e.g., Hofstede et al. (2010), p. 486]. When, however, as the authors state, national wealth is controlled for, the correlation weakens and becomes −0.36. Some researchers identify the dimensions as separate and others as one. For this study, we use the dimensions separately. In addition to using the cultural values of Hofstede, we also included the cultural values of Schwartz. Schwartz states (1994, p. 117) that when compared to Hofstede's dimensions his ideal value types are different based on different “theoretical reasoning, different methods, a different set of nations, different types of respondents, data from a later historical period, a more comprehensive set of values, and value items screened to be reasonably equivalent in meaning across cultures.” Whereas, Hofstede assumes four (he later added a fifth dimension Long-Term versus Short-Term Orientation and a sixth dimension Indulgence vs. Restraint) value dimensions: Individualism-Collectivism, Power Distance, Uncertainty Avoidance, and Masculinity-Femininity, Schwartz (2020) identified three bipolar value dimensions: autonomy (intellectual and affective) vs. embeddedness, egalitarianism vs. hierarchy, and harmony versus mastery.

It is noteworthy, though, that empirically, given all the differences, the autonomy (intellectual and affective) vs. embeddedness dimension is similar to the Individualism-Collectivism dimension, as Schwartz noted himself (1994, p. 117). The egalitarianism vs. hierarchy dimension is similar to Hofstede's Power Distance dimension. Hierarchy refers to assuming submissiveness from people below (Schwartz, 2020). Alternatively, low Power Distance or Egalitarianism is society's appreciation of individuals as equals and sharing of interests fairly (Schwartz, 2020). Despite their similarities, cultural values identified by Hofstede and Schwartz are treated as separate constructs.

## Methods

### Participants, Instruments, and Data Sources

The current study used secondary data that were collected in two large-scale, global studies of cultural values that were conducted across 76 countries and regions by Hofstede (2001) and Hofstede et al. (2010) and across 75 countries by Schwartz (2020), with a combined sample size of over 150,000 participants. The present study did not consider data for all the countries and regions because either no COVID-19 death numbers were available for some of these countries and regions or the studies had reported subsamples within countries (e.g., Hofstede's combined scores for Arab countries or East African countries or Schwartz's separation of scores for Israeli Muslims and Israeli Jews). Some of the studies also reported subsamples for countries such as Canada, Germany, Israel, and Switzerland, but we only considered the overall country score. In the case of both studies, we only included data for values that reflected, or were most closely related to, Individualism-Collectivism and Power Distance.

Additionally, data on confirmed coronavirus cases and deaths were retrieved from Johns Hopkins University (2020) and from the World Health Organization (2020). For the correlational analysis, the results were based on 70 countries from the Hofstede study (survey participants were IBM employees) and 74 countries from the Schwartz study (survey participants were school teachers, undergraduate students, adolescents, and adults; see Table 1).

**Table 1:** Descriptive statistics for study variables in the 88 countries.

**Country**	**Deaths July**	**Deaths Dec**	**Median age**	**IHDI**	**Gov't Eff**	**GDP per capita**	**Gini index**	**BMI**	**Health c exp**	**Pop dens**	**IndivH**	**Power DistH**	**Intel AutonS**	**Affect AutonS**	**Embed-dednS**	**EgalitS**	**Hier-archyS**
Argentina	4.42	939	31.5	0.714	−0.09	10,006	41.4	27.7	1,990	16.52	46	49	4.34	3.73	3.52	4.96	2.1
Australia	0.44	36	37.9	0.862	1.57	54,907	34.4	27.2	5,005	3.32	90	38	4.35	3.86	3.59	4.79	2.29
Austria	8.01	639	43.5	0.843	1.49	50,277	29.7	25.4	5,879	109.3	55	11	4.9	4.29	3.11	4.89	1.75
Bangladesh	1.5	45	27.6	0.465	−0.74	1,909	32.4	21	110	1265	20	80					
Belgium	85.69	1656	41.9	0.849	1.03	46,117	27.4	25.5	5,405	382.7	75	65	4.64	3.94	3.25	5.2	1.69
Bolivia	16.72	778	25.6	0.533	−0.7	3,552	42.2	25.9	496	10.78			4.34	2.71	4.07	4.74	2.66
Bosnia Herzegowina	6.8	1196	43.1	0.658	−0.63	6,073	33	26.1	1,301	64.33			4.18	3.3	4.01	4.66	1.73
Brazil	35.39	896	33.5	0.574	−0.19	8,717	53.9	25.9	1,531	25.43	38	69	4.27	3.52	3.62	4.89	2.37
Bulgaria	4.03	715	44.6	0.714	0.34	9,738	40.4	26	1,634	64.01	30	70	4.29	3.47	3.87	4.13	2.68
Canada	23.87	392	41.1	0.841	1.73	46,195	33.8	27.2	5,200	4.15	80	39	4.58	4.08	3.38	4.85	2.03
Chile	37.74	858	35.3	0.696	1.06	14,897	44.4	27.8	2,306	25.71	23	63	4.32	3.03	3.64	5.06	2.25
China (comb)	0.33	3	38.4	0.636	0.52	10,262	38.5	23.9	935	153.3	20	80	4.18	3.3	3.74	4.23	3.49
Colombia	11.66	819	31.3	0.585	0.07	6,432	50.4	25.9	1,155	45.86	13	67	4.3	3.61	3.86	4.69	2.9
Costa Rica	0.74	409	33.5	0.645	0.42	12,238	48	26.9	1,337	99.77	15	35	4.37	3.49	3.49	4.85	2.29
Croatia	2.93	894	44.3	0.768	0.41	14,853	30.4	25.5	1,876	73.36	33	73	4.35	3.92	4	4.6	2.55
Cyprus	1.6	92	37.3	0.788	0.99	27,858	31.4	27	2,625	130.7			3.83	3.21	4.04	4.85	1.96
Czech Republic	3.34	1,031	43.2	0.85	0.89	23,495	24.9	26.9	3,041	138.6	58	57	4.62	3.49	3.59	4.45	2.22
Denmark	10.52	199	42.3	0.873	1.94	59,822	28.7	25.3	5,794	136.5	74	18	4.77	4.3	3.19	5.03	1.86
Ecuador	30.03	793	27.9	0.607	−0.4	6,184	45.4	27	955	71.04	8	78					
Egypt	4.07	72	24.6	0.492	−0.42	3,020	31.5	29.2	614	102.8			3.9	2.5	4.45	4.42	2.2
El Salvador	4.33	200	27.6	0.521	−0.47	4,187	38.6	27.4	592	313	19	66					
Estonia	5.22	154	42.4	0.818	1.17	23,660	30.4	25.5	2,428	31.29	60	40	4.23	3.36	3.81	4.58	2.04
Ethiopia	0.13	17	19.5	0.337	−0.63	858	35	20.6	67	115			3.94	2.61	4.54	4.4	2.33
Finland	5.96	95	43.1	0.876	1.93	48,686	27.4	25.9	4,457	18.23	63	33	4.93	3.96	3.37	4.9	1.8
France	44.83	953	42.3	0.809	1.38	40,494	31.6	25.3	5,250	119.2	71	68	5.13	4.39	3.2	5.05	2.21
Georgia	0.4	596	38.3	0.692	0.83	4,769	36.4	27.2	796	57.41			4	3.47	4.12	4.66	2.46
Germany	10.95	355	45.7	0.861	1.59	46,259	31.9	26.3	6,098	240.4	67	35	4.85	4.19	3.09	5.01	1.82
Ghana	0.47	11	21.5	0.427	−0.21	2,202	43.5	24.2	168	136.6			3.89	2.49	4.27	4.73	2.68
Great Britain/UK	67.76	1,037	40.5	0.845	1.44	42,300	34.8	27.3	4,620	280.6	89	35	4.62	4.26	3.34	4.92	2.33
Greece	1.8	437	45.6	0.766	0.41	19,583	34.4	27.3	2,340	80.86	35	60	4.39	3.92	3.41	4.84	1.83
Guatemala	7.55	266	22.9	0.472	−0.68	4,620	48.3	26.5	483	167.2	6	95					
Hong Kong, China			44.8	0.815	1.74	48,756				7140	25	68	4.28	3.2	3.76	4.5	2.91
Hungary	6.09	937	43.3	0.777	0.5	16,476	30.6	26.3	2,115	106.7	80	46	4.57	3.63	3.6	4.51	1.94
India	1.8	107	28.4	0.477	0.17	2,104	37.8	21.9	275	464.1	48	77	4.02	3.48	3.97	4.45	3.05
Indonesia	1.39	77	29.7	0.584	0.18	4,136	39	22.9	375	151	48	78	3.94	3.41	4.27	4.32	2.56
Iran	16.15	650	32	0.706	−0.55	5,701	40.8	26.2	1,691	51.58	48	58	3.96	2.97	4.18	4.53	3.23
Ireland	35.97	446	38.2	0.865	1.28	78,661	32.8	27.5	5,897	71.68	48	28	4.54	4.05	3.41	4.9	2.09
Israel	4.18	363	30.5	0.809	1.33	43,641	39	26.3	3,207	400	48	13	4.53	3.77	3.63	4.76	2.51
Italy	57.89	1,185	47.3	0.776	0.46	33,190	35.9	26	3,624	205.6	76	50	4.91	3.3	3.46	5.27	1.6
Jamaica	0.34	99	30.7	0.604	0.5	5,582	45.5	27.4	559	273.4	39	45					
Japan	0.78	25	48.4	0.882	1.59	40,247	32.9	22.6	4,504	346.9	46	54	4.78	3.76	3.49	4.36	2.65
Jordan	0.1	365	23.8	0.617	0.1	4,330	33.7	28.9	738	114.9			4.05	3.36	4.2	4.4	2.5
Latvia	1.61	273	43.9	0.776	1.11	17,836	35.6	25.8	1,896	30.33	70	44	4.22	3.48	3.83	4.32	1.8
Lithuania	2.83	461	45.1	0.775	1.04	19,456	37.3	26.6	2,313	43.44	60	42					
Luxembourg	18.26	751	39.7	0.822	1.73	114,705	34.9	26.5	6,048	241.7	60	40					
Malaysia	0.39	14	30.3		1	11,415	41	25.3	1,194	98.51	26	104	4.15	2.98	4.35	4.41	2.25
Malta	1.86	467	42.6	0.815	0.86	29,416	29.2	27.2	3,897	1380	59	56					
Mexico	28.79	945	29.2	0.595	−0.16	9,863	45.4	28.1	1,066	66.33	30	81	4.36	2.83	3.9	4.73	2.13
Morocco	0.71	195	29.5		−0.12	3,204	39.5	25.6	467	82.7	46	70					
Namibia		74	21.8	0.417	0.1	4,958	59.1	24.3	883	3.09			4.03	3.29	4.04	4.48	2.53
Nepal	0.14	62	24.6	0.43	−1.05	1,071	32.8	22.2	180	203.3			4.07	2.99	4.18	4.63	3.03
Netherlands	35.71	640	43.3	0.87	1.8	52,448	28.5	25.4	5,635	508.2	80	38	4.85	4.13	3.19	5.03	1.91
New Zealand	0.45	5	38	0.836	1.67	42,084		27.9	4,024	18.31	79	22	4.65	4.21	3.27	4.94	2.27
Nigeria	0.38	6	18.1	0.349	−1.09	2,230	35.1	23.4	233	226.3			3.66	2.54	4.41	4.79	2.72
North Macedonia	18.68	1,165	39.1	0.66	0	6,093	34.2		1,073	82.61			4.24	3.01	3.91	4.4	2.72
Norway	4.76	78	39.8	0.889	1.86	75,420	27	26	6,818	14.84	69	31	4.68	3.69	3.45	5.12	1.49
Oman	5.65	292	30.6	0.725	0.26	15,474		26.9	1,730	16.5			3.73	2.87	4.5	4.49	2.15
Pakistan	2.54	44	22.8	0.386	−0.68	1,349	33.5	23.8	178	286.5	14	55	3.76	3.11	4.31	4.65	2.44
Panama	22.98	870	29.7	0.626	0.07	15,731	49.2	27.1	1,857	58.04	11	95					
Peru	38.23	1,133	31	0.612	−0.07	6,978	42.8	26.3	767	25.76	16	64	4.3	2.98	3.92	4.84	2.76
Philippines	1.5	83	25.7	0.582	0.05	3,485	44.4	23.2	394	367.5	32	94	3.95	3	4.03	4.59	2.68
Poland	4.18	717	41.7	0.801	0.6	15,595	29.7	26.4	2,015	123.6	60	68	4.31	3.32	3.86	4.48	2.51
Portugal	16.22	643	46.2	0.742	1.15	23,145	33.8	26.2	3,242	111.3	27	63	4.53	3.62	3.43	5.21	1.89
Romania	9.92	785	43.2	0.725	−0.28	12,920	36	25.3	1,576	83.58	30	90	4.61	3.45	3.78	4.48	2
Russian Federation	8.03	375	39.6	0.743	0.15	11,585	37.5	26.5	1,488	8.91	39	93	4.3	3.51	3.81	4.38	2.72
Senegal	0.95	23	18.5	0.347	−0.06	1,447	40.3	23	146	86.97			3.89	2.39	4.45	4.92	2.63
Serbia	5.99	428	41.6	0.685	0.02	7,402	36.2	25.8	1,485	99.9	25	86	4.72	3.7	3.57	4.44	1.61
Singapore	0.48	5	42.2	0.81	2.22	65,233		23.7	4,439	8358	20	74	3.86	3.3	4	4.6	2.82
Slovakia	0.51	325	41.2	0.804	0.67	19,329	25.2	26.5	2,180	113.5	52	104	4.29	2.99	3.82	4.58	2
Slovenia	5.37	1217	44.5	0.858	1.08	25,739	24.2	26.9	3,158	103.2	27	71	4.88	3.72	3.71	4.56	1.62
South Africa	7.52	447	27.6	0.463	0.37	5,520	63	27.3	1,129	48.89	65	49	3.85	3.48	4.03	4.52	2.59
South Korea	0.56	16	43.7	0.777	1.38	31,762	31.6	23.9	3,214	527.3	18	60	4.22	3.46	3.68	4.42	2.9
Spain	60.8	1,066	44.9	0.765	1	29,614	34.7	26.7	3,576	93.74	51	57	4.99	3.67	3.31	5.23	1.84
Suriname	3.13	203	29	0.557	−0.59	6,855	57.6	27.4	1,180	3.76	47	85					
Sweden	54.45	820	41.1	0.874	1.83	51,610	28.8	25.8	5,828	24.61	71	31	5.09	4.24	3.12	4.9	1.83
Switzerland	23.11	752	43.1	0.882	1.95	81,994	32.7	25.3	8,114	219	68	34	4.83	4.26	3.27	4.96	2.33
Taiwan, China	0.03		42.5		1.44		33.8			672.6	17	58	4.36	3.27	3.82	4.31	2.69
Thailand	0.08	1	40.1	0.635	0.36	7,808	36.4	24.1	723	136.6	20	64	4.02	3.63	4.02	4.29	3.23
Trinidad and Tobago	0.58	89	36.2		0.1	17,277	40.3	28.7	2,100	272.8	16	47					
Turkey	6.56	233	31.5	0.675	0.05	9,043	41.9	27.8	1,171	109.6	37	66	4.45	3.37	3.77	4.77	2.97
Uganda		5	16.7	0.387	−0.59	777	42.8	22	139	228.9			3.8	2.68	4.23	4.39	2.99
Ukraine	3.2	406	41.2	0.701	−0.3	3,659	26.1	26	683	75.49			4.08	3.49	3.93	4.31	2.56
United States	41.71	991	38.3	0.797	1.49	65,281	41.4	28.5	10,624	36.19	91	40	4.19	3.87	3.67	4.68	2.37
Uruguay	0.9	41	35.8	0.703	0.7	16,190	39.7	26.8	2,169	19.85	36	61					
Venezuela	0.33	36	29.6	0.6	−1.66		46.9	27.2	384	32.24	12	81	4.44	3.26	3.74	4.77	2.09
Viet Nam		0	32.5	0.58	0.04	2,715	35.7	21.6	440	313.9	20	70					
Yemen		20	20.2	0.316	−2.28	968	36.7	25.8		56.49			3.68	2.44	4.63	4.73	2.28
Zimbabwe	0.14	23	18.7	0.435	−1.21	1,464	44.3	23.4	198	38.42			3.62	3.62	3.62	3.62	3.62

*Deaths per 100 k as of July 14, 2020 JH; Deaths per 100 k as of Dec. 29, 2020 WHO. IHDI stands for inequality-adjusted Human Development Index, “H” stands for Hofstede, “S” stands for Schwartz (for more details to each variable, see Method section)*.

#### Hofstede

Cultural values reported by Hofstede were based on his survey conducted in the multinational corporation IBM between 1967 and 1973 with more than 116,000 respondents from 72 countries in 20 languages. Additional research and country scores were added and updated (Hofstede et al., 2010) and used in the current study. The results showed the statistical independence of the four initial value dimensions of Individualism-Collectivism, Power Distance, Uncertainty Avoidance, and Masculinity-Femininity. Later, two other dimensions were added: Long-term versus Short-term orientation and Indulgence versus Restraint. For the current study, we only included the value dimensions of Individualism-Collectivism and Power Distance. These two dimensions have been replicated in other studies as well (e.g., Van Nimwegen, 2002).

In Individualism-Collectivism, “Individualism stands for a society in which the ties between individuals are loose: everyone is expected to look after him/herself and his/her immediate family only. Collectivism stands for a society in which people from birth onward are integrated into strong, cohesive in-groups, which throughout people's lifetime continue to protect them in exchange for unquestioning loyalty” (Hofstede, 2001, p. 225). Individualism and Collectivism were assessed using 14 work–goal-related questions.

Power Distance is “the extent to which the less powerful members of institutions and organizations within a country expect and accept that power is distributed unequally” (Hofstede, 2001, p. 98). Power Distance was assessed using three items.

#### Schwartz

Cultural values reported by Schwartz (2020) were based on data collected between 1988 and 2002 from 233 samples from 68 countries. In total, there were 67,145 participants. The samples were obtained through convenience sampling and included school teachers, undergraduate students, adolescents, and adults. Schwartz distinguished three value dimensions: Autonomy (Affective and Intellectual) versus Embeddedness, Egalitarianism vs. Hierarchy, and Harmony vs. Mastery [see also Schwartz and Boehnke (2004)]. These values were assessed using the Schwartz Value Survey, which included 56 or 57 value items (SVS:12). The SVS presents two lists of abstract value items. The first list contains 30 items describing potentially desirable end-states in noun form (e.g., equality) including a short explanation (“EQUALITY-equal opportunity for all”). The second list contains 26 or 27 items that describe potentially desirable ways of acting in adjective form. Participants rated the importance of each value item “as a guiding principle in MY life” on a 9-point scale where 7 = of supreme importance, while−1 = opposition to my values. In order to conduct cross-cultural comparisons, multidimensional scaling analyses were conducted to ensure that the meaning of the items was relatively similar across cultures. We included the following five values in our analyses: Affective Autonomy, Intellectual Autonomy (which is relatively similar to Hofstede's Individualism), Embeddedness (which is similar to Hofstede's Collectivism), Egalitarianism (which is similar to low Power Distance), and Hierarchy (which is similar to high Power Distance).

**Intellectual Autonomy**: In cultures with high Intellectual Autonomy, people are viewed as autonomous, bounded entities. They are encouraged to express their own preferences, feelings, and ideas. “Intellectual autonomy encourages individuals to pursue their own ideas and intellectual directions independently” (Schwartz, 2020).

**Affective Autonomy**: “Affective autonomy encourages individuals to pursue arousing, affectively positive personal experience” (Schwartz, 2020).

**Embeddedness**: In embedded cultures, people are viewed as entities embedded in collectivity. Meaning in life is expected to be derived largely through “social relationships, through identifying with the group, participating in its shared way of life, and striving toward its shared goals” (Schwartz, 2020).

**Egalitarianism**: “Egalitarian cultures seek to induce people to recognize one another as moral equals who share basic interests as human beings. They try to socialize their members to internalize a commitment to cooperate and to feel concern for everyone's welfare” (Schwartz, 2020).

**Hierarchy**: Hierarchy cultures rely on hierarchical systems of ascribed roles. “They define the unequal distribution of power, roles, and resources as legitimate and even desirable. People are socialized to take a hierarchical distribution of roles for granted to comply with the obligations and rules attached to their roles, to show deference to superiors, and to expect deference from subordinates” (Schwartz, 2020).

#### COVID-19 Confirmed Cases and Deaths

**Johns Hopkins University**: Johns Hopkins University JHU maintains a website that reports daily confirmed coronavirus cases, coronavirus deaths, fatality rate, and combined coronavirus deaths per 100,000 population for 164 countries (https://coronavirus.jhu.edu/data/mortality). We used coronavirus deaths reported for July 14, 2020.

**World Health Organization**: The World Health Organization collects and reports data from 216 countries and territories related to the coronavirus pandemic. Every day, it releases a situation report with data on the confirmed total coronavirus cases, confirmed new cases, total deaths, total new deaths, and transmission classification (https://www.who.int/emergencies/diseases/novel-coronavirus-2019/situation-reports). We used COVID-19 related deaths per 1 million population reported for December 29, 2020, as there were no reports of deaths provided during summer 2020.

The reason why we used COVID-19 deaths data from the WHO and from JHU is that, before August 27, 2020, the WHO only reported absolute numbers of deaths and not relative numbers according to population. The reason why we did not use data from JHU for December is a change in their data presented on their website not allowing us to search for a specific date anymore. Ultimately, both WHO and JHU data should be identical, although there is no way for us to verify.

#### Control Variables

**Inequality-adjusted Human Development Index IHDI**: The IHDI represents a national average of human development combining the three dimensions of health, education, and income; it also accounts for within-country differences in the three dimensions, as provided by the United Nations Development Programme (2020). The range in our study was from 0.32 to 0.89 (*M* =0.68, *SD* =0.16, *N* = 84). The higher the IHDI, so we predicted, the lower would be the COVID-19 death rate.

**Government effectiveness**: Government effectiveness is one of six worldwide governance indicators relevant to our current study. Government effectiveness captures “perceptions of the quality of public services, the quality of the civil service and the degree of its independence from political pressures, the quality of policy formulation and implementation, and the credibility of the government's commitment to such policies.” (Kaufmann et al., 2010, p. 4). The range can be from −2.5 to + 2.5. For our sample of 88 countries, it was from −2.28 to 2.22 (*M* = 0.47, *SD* = 0.93). It might indicate how effective governments implement public health policies related to COVID-19. The higher the government effectiveness, so we predicted, the lower would be the COVID-19 death rate.

**Gross domestic product GDP per capita**: The GDP per capita is the purchasing power parity PPP of all goods and services produced within a country in a given year divided by the population for the same year. It takes into account relative costs of living and inflation and is therefore also an indicator of a country's standard of living. We used the GDP per capita data of the World Bank (2019). We predicted that the higher the GDP per capita, the easier it would be for a country to finance measures to fight COVID-19 and the lower would ultimately be the COVID-19 related death rate.

**Gini Index**: The Gini index developed by Corrado Gini is a measure of income inequality (Giorgi and Gigliarano, 2017). The Gini coefficient can vary between 0 and 100%, where 0% stands for perfect equality with everyone having the same income and 100% stands for maximal inequality with a few having almost all income and almost everyone else having almost no income. We report the data of the World Bank (2021). We predict that the number of coronavirus deaths will be smaller in countries with income equality compared to countries with high income inequality.

**Health care expenses per capita**: This is a measure indicating how much money, both public and private, is spent for health per capita. We refer to data from the World Health Organization (World Health Organization, 2018). It shows total health expenditure per capita in 2018 PPP inflation-adjusted U.S. dollars. We predict that high expenses could help prevent COVID-19 related deaths.

**Population density**: We also included population density per square km as a potential variable linked to the spreading of the coronavirus. We used the data from the United Nations (United Nations, Department of Economic and Social Affairs, 2019). Although some studies show a positive relationship between population density and COVID-19 deaths [e.g., Zhang and Schwartz (2020) within the United States], other studies do not show such a relationship (e.g., Carozzi et al., 2020).

**Median age**: We included the median age for 2020 provided by the United Nations, Department of Economic and Social Affairs (2020). The range was from 16.7 to 48.4 years (*M* = 35.4, *SD* = 8.4, *N* = 88). To check its validity, we correlated this median age with the median age provided by the CIA Worldfactbook for 2018. The correlation for the 86 countries (two missing: Hong Kong and Taiwan) was 0.987, *p* < 0.001. We predicted that the higher the median age, the higher would be the COVID-19 death rate [see also Wang X. Q. et al. (2020) and Li et al. (2021)].

**Body Mass Index BMI**: The BMI is defined by the body weight in kilograms divided by the square of the body height in meters (kg/m^2^). A BMI below 18.5 indicates underweight, a BMI greater than 30 indicates obesity. We included BMI overall means per country from the World Health Organization (World Health Organization, 2014) since some studies have shown obesity to be a high-risk factor of COVID-19 deaths (e.g., Fakhry AbdelMassih et al., 2020; Tartof et al., 2020).

The control variables we included refer to two combined variables (IHDI and government effectiveness), three economic variables of countries (GDP, Gini index, and Health care expenses per capita), one socio-geographic variable (population density), and two individual-biological/physiological variables (median age and Body Mass Index).

### Procedure/Data Analysis

We combined the data about the confirmed coronavirus cases and deaths, as reported by the Johns Hopkins University and the World Health Organization for each country (accessed on July 14, 2020), with all the control variables and the Individualism-Collectivism and Power Distance values reported by Hofstede and Schwartz (see Table 1; for descriptive statistics of all variables see Table 2), and then conducted Pearson correlations for all measures (see Table 3). Since the number of confirmed COVID-19 cases is highly dependent on the extent of testing, we only used the number of COVID-19 related deaths for further analyses.

**Table 2:** Descriptive statistics for all variables.

		***n***	***M***	***SD***
1	Deaths July	83	12.10	17.91
2	Deaths Dec	86	442.58	405.97
3	IHDI	84	0.68	0.16
4	Government effectivn.	88	0.47	0.93
5	GDP per capita	86	21920.26	23153.10
6	Gini index	84	37.25	7.70
7	Health care expenses	85	2370.91	2159.41
8	Population dens.	88	335.93	1162.01
9	Median age	88	35.35	8.40
10	BMI	85	25.80	1.83
11	IndividualismH	70	44.21	23.47
12	PowerDistanceH	70	58.99	21.74
13	IntelAutonomyS	74	4.32	0.37
14	AffectAutonomyS	74	3.45	0.50
15	EmbeddednessS	74	3.79	0.38
16	EgalitarianismS	74	4.67	0.29
17	HierarchyS	74	2.36	0.47

**Table 3:** Pearson correlations of COVID-19 deaths, control variables, and cultural values.

		**1**	**2**	**3**	**4**	**5**	**6**	**7**	**8**	**9**	**10**	**11**	**12**	**13**	**14**	**15**	**16**
1	Deaths July																
2	Deaths Dec	0.70^***^															
3	IHDI	0.30^**^	0.38^***^														
4	Governmt eff	0.25^*^	0.16	0.84^***^													
5	GDP	0.35^***^	0.18	0.74^***^	0.81^***^												
6	Gini index	−0.04	−0.12	−0.54^***^	−0.42^***^	−0.41^***^											
7	Health c exp	0.46^***^	0.31^**^	0.78^***^	0.82^***^	0.92^***^	−0.41^***^										
8	Pop dens	−0.08	−0.14	0.12	0.26^*^	0.24^*^	−0.24	0.11									
9	Median age	0.24^*^	0.43^***^	0.89^***^	0.71^***^	0.55^***^	−0.54^***^	0.62^***^	0.15								
10	BMI	0.20	0.39^***^	0.41^***^	0.19	0.20	0.08	0.27^*^	−0.20	0.27^*^							
11	IndividualismH	0.35^**^	0.23	0.63^***^	0.57^***^	0.56^***^	−0.39^***^	0.68^***^	−0.17	0.43^***^	0.19						
12	Power DistanceH	−0.15	−0.04	−0.55^***^	−0.60^***^	−0.60^***^	0.31^*^	−0.62^***^	0.10	−0.34^**^	−0.23	−0.59^***^					
13	Intel AutonomyS	0.48^***^	0.48^***^	0.74^***^	0.60^***^	0.62^***^	−0.41^***^	0.65^***^	−0.11	0.70^***^	0.29^*^	0.43^***^	−0.42^***^				
14	Affect AutonomyS	0.33^**^	0.29^*^	0.74^***^	0.67^***^	0.68^***^	−0.30^*^	0.73^***^	−0.06	0.66^***^	0.26^*^	0.60^***^	−0.65^***^	0.72^***^			
15	EmbeddednessS	−0.48^**^	−0.39^***^	−0.76^***^	−0.66^***^	−0.70^***^	0.29^*^	−0.73^***^	0.04	−0.69^***^	−0.29^*^	−0.53^***^	0.62^***^	−0.86^***^	−0.86^***^		
16	EgalitarianismS	0.56^***^	0.35^**^	0.34^**^	0.34^**^	0.49^***^	−0.14	0.51^***^	−0.07	0.23^*^	0.31^**^	0.38^**^	−0.46^***^	0.54^***^	0.31^**^	−0.48^***^	
17	HierarchyS	−0.31^**^	−0.35^**^	−0.49^***^	−0.34^**^	−0.42^***^	0.41^***^	−0.47^***^	0.20	−0.45^***^	−0.42^***^	−0.43^***^	0.31^*^	−0.58^***^	−0.36^**^	0.44^***^	−0.59^***^

*“H” stands for Hofstede values; “S” stands for Schwartz values.*

* *p < 0.05,*

** *p < 0.01,*

*** *p ≤ 0.001*.

Analyzing the Pearson Correlations of the control variables for values higher than 0.75 and thus for possible multicollinearity (see Table 3), it becomes clear that IHDI and government effectiveness correlate highly with each other and with other control variables such as median age or GDP. This is not surprising since IHDI and government effectiveness are a combination of other variables. Additionally, GDP per capita correlates highly with health care expenses per capita (0.92). Considering these high correlations and considering that it is difficult to interpret the combined variables, we excluded the three control variables IHDI, government effectiveness, and healthcare expenses per capita from the regression analyses.

Among the cultural values, there are only three correlations higher than 0.72, namely, between intellectual autonomy, affective autonomy, and embeddedness. Since from a theoretical perspective, affective autonomy, seems least relevant, we decided to exclude it from the regression analyses to avoid multicollinearity.

For the hierarchical regression analyses (see Tables 4, 5) we entered in Step 1 the five “control” variables: GDP per capita, Gini index, Population density, Median age, and BMI. In Step 2, we included the six cultural values; two of Hofstede (Individualism and Power Distance) and four of Schwartz (Intellectual Autonomy, Embeddedness, Egalitarianism, and Hierarchy). COVID-19 related deaths were not normally distributed variables, so we ran bootstrapped analyses with 1,000 samples.

**Table 4:** Hierarchical regression results for COVID-19 deaths per 100,000 people on July 14, 2020 (JH).

**Variable**	***B***	**95% CI**	**for B**	**SE B**	**β**	***R*^**2**^**	**Δ*R*^**2**^**
		**LL**	**UL**				
Step 1: Control variables						0.23^*^	
Constant	−163.42	−297.08	−34.15	69.14			
GDP per capita	0.00	0.00	0.00	0.00	0.31^†^		
Gini index	0.58	−0.48	1.78	0.48	0.21		
Pop density	0.04	−0.03	0.12	0.03	0.27		
Median Age	0.44	−0.68	1.82	0.57	0.14		
BMI	4.96	0.56	9.27	2.35	0.34^†^		
Step 2: Control variables and cultural values						0.56^***^	0.33^***^
Constant	−473.102	−794.08	−230.23	169.77			
GDP per capita	0.00	0.00	0.00	0.00	0.04		
Gini index	0.62	−0.25	1.82	0.45	0.23		
Pop density	0.03	−0.03	0.08	0.03	0.16		
Median Age	0.39	−1.06	2.02	0.71	0.12		
BMI	1.91	−2.19	6.10	2.33	0.13		
IndividualismH	0.32	0.05	0.62	0.14	0.35^*^		
Power DistanceH	0.30	−0.04	0.64	0.17	0.32^†^		
Intel AutonomyS	18.49	−18.49	59.53	17.53	0.30		
EmbeddednessS	10.48	−29.05	55.31	18.26	0.16		
EgalitarianismS	47.64	21.28	80.68	14.12	0.66^***^		
HierarchyS	9.14	−2.20	23.22	6.77	0.21		

*Bootstrapped results for 1,000 samples; CI, BCa Confidence interval; LL, lower limit; UL, upper limit. “H” stands for Hofstede values, “S” stands for Schwartz values*.

† *p < 0.07,*

* *p < 0.05,*

*** *p ≤ 0.001*.

**Table 5:** Hierarchical regression results for COVID-19 deaths per 1 million on December 29, 2020 (WHO).

**Variable**	***B***	**95% CI**	**for B**	**SE B**	**β**	***R*^**2**^**	**Δ*R*^**2**^**
		**LL**	**UL**				
Step 1: Control variables						0.30^**^	
Constant	−4746.97	−7024.09	−2697.36	1163.80			
GDP per capita	−0.00	−0.01	0.00	0.00	−0.18		
Gini index	1.52	−18.42	23.15	9.32	0.03		
Pop density	0.72	−0.23	1.93	0.60	0.24		
Median Age	17.72	−3.99	44.26	10.23	0.28		
BMI	175.83	84.33	267.58	44.26	0.61^***^		
Step 2: Control variables and cultural values						0.49^**^	0.19^*^
Constant	−11512.48	−18511.22	−4917.24	3784.25			
GDP per capita	−0.01	−0.02	0.00	0.01	−0.37		
Gini index	5.74	−14.30	28.56	10.32	0.11		
Pop density	0.61	−0.35	1.85	0.51	0.20		
Median Age	13.96	−8.32	44.82	12.55	0.22		
BMI	138.02	42.31	241.71	53.22	0.48^**^		
IndividualismH	3.70	−0.72	7.72	2.53	0.21		
Power DistanceH	2.57	−3.90	9.08	3.79	0.14		
Intel AutonomyS	677.17	−144.52	1587.65	392.29	0.55^*^		
EmbeddednessS	449.76	−610.91	1491.86	408.74	0.35		
EgalitarianismS	563.82	58.37	1197.13	284.74	0.40^*^		
HierarchyS	91.07	−249.71	418.51	174.12	0.11		

*Bootstrapped results for 1,000 samples; CI, BCa Confidence interval; LL, lower limit; UL, upper limit. “H” stands for Hofstede values, “S” stands for Schwartz values*.

* *p < 0.05,*

** *p < 0.01,*

*** *p ≤ 0.001*.

## Results

The first hypothesis stated that individualism would be positively related to coronavirus deaths, while collectivism would be negatively related to coronavirus deaths. Hofstede saw individualism-collectivism as a continuum of one dimension; Schwartz assessed these values with different dimensions. Additionally, we predicted that high power distance or hierarchy would be negatively related to coronavirus deaths, and low power distance or egalitarianism would be positively correlated to coronavirus deaths, while we control for the influence of the five control variables.

As of July 14, 2020, the mean number of coronavirus deaths per 100,000 people was 12.10 (*SD* = 17.91) as per the data from the Johns Hopkins University. As of December 29, 2020, the mean number of coronavirus deaths per 1 million people was 442.58 (*SD* = 405.97) as per the data from the World Health Organization (see Tables 1, 2 for data and descriptive statistics of all variables). The World Health Organization did not provide data on coronavirus deaths per population during the summer of 2020. The number of COVID deaths from July correlates highly with the number of deaths from December, *r* = 0.70, *p* < 0.001.

### Correlational Analyses

The number of deaths in July correlated positively and significantly with GDP and median age. These deaths correlated significantly and positively with individualism values across all countries and all measures (see Table 3). The number of deaths in July also correlated positively and significantly with Hofstede's Individualism, with Schwartz's Intellectual Autonomy, and with Schwartz's Affective Autonomy. When collectivism values were assessed using Schwartz's scale (Embeddedness), the coronavirus deaths correlated significantly and negatively with collectivism values across all countries.

The results for Power Distance were not as consistent. As predicted, the coronavirus deaths were significantly and negatively correlated with Schwartz's Hierarchy, and—as predicted—significantly and positively correlated with Schwartz's Egalitarianism. However, the deaths did not correlate significantly with Hofstede's Power Distance. Controlling Hofstede's I/C for Hofstede's PDI, and *vice versa*, does not affect the results.

The number of deaths in December correlated positively and significantly with median age and BMI (see Table 3). These December deaths correlated significantly and positively with all the same values as during July, except that the correlation between Individualism Hofstede and the number of deaths was now 0.23, *p* = 0.06.

### Hierarchical Regression Analyses

We then conducted hierarchical regression analyses once for the COVID-19 deaths on July 14, 2020, from the Johns Hopkins University data (see Table 4) and once for the deaths on December 29, 2020, from the World Health Organization data (see Table 5). For July, the five control variables entered together in Step 1 predict COVID-19 related deaths significantly, *F*_(5, 45)_ = 2.74, *p* = 0.03, explaining 23% of the variance in deaths. GDP and BMI were marginally significant predictors of COVID-19 deaths. The cultural values added together in Step 2, significantly predict COVID-19 related deaths, *F*_(6, 39)_ = 4.88, *p* = 0.001, explaining an additional 33% of the variance in deaths. The three values, Individualism H, Egalitarianism S, and Power Distance H (marginally), were significant predictors. The overall model explained 56.2% of the variance in COVID-19 deaths, *F*_(11, 39)_ = 4.55, *p* < 0.001.

For December, the five control variables entered together in Step 1 predict COVID-19 related deaths significantly, *F*_(5, 45)_ = 3.86, *p* = 0.005, explaining 30% of the variance in deaths (see Table 5). Only BMI was a significant predictor of COVID-19 deaths. The cultural values added together in Step 2, significantly predict COVID-19 related deaths, *F*_(6, 39)_ = 2.44, *p* = 0.04, explaining an additional 19% of the variance in deaths. The two values, Intellectual Autonomy S and Egalitarianism S were significant predictors. The overall model explained 49.1% of the variance in COVID-19 deaths, *F*_(11, 39)_ = 3.42, *p* = 0.002.

Results of both hierarchical regression analyses were relatively consistent. Considering both models, among the five control variables GDP and BMI are the strongest predictors of COVID-19 deaths. The values, Individualism H, Power Distance H, Intellectual Autonomy S, and Egalitarianism S were significant predictors.

## Discussion

With news of some countries being able to flatten the curve and contain the coronavirus infections and some others living in great uncertainty and dread because of rising coronavirus infections, the goal of this study was to investigate whether the confirmed coronavirus deaths relate to the cultural values of Individualism-Collectivism/Autonomy-Embeddedness and Power-Distance/Hierarchy-Egalitarianism. In order to acknowledge different countries' varying levels of development and differences in demographics, we controlled for the two economic variables: GDP and Gini index; for one socio-geographic variable: population density; and for two individual health factors: median age and BMI. Findings of the regression analyses show that of the five control variables, only GDP and BMI were significant. The higher the BMI, the higher the number of COVID-19 related deaths. This finding is supported by Fakhry AbdelMassih et al. (2020) and Pettit et al. (2020), who found that obesity is a potent predictor of death from COVID-19: as the BMI increases, the risk for mortality also increases.

It is surprising that the higher the GDP per capita, the higher the number of COVID-19 related deaths. One potential explanation is that people from more affluent countries travel more across the world (are more mobile and can afford lifestyles that support the spread, such as eating at restaurants) and are therefore more likely to get infected and spread the coronavirus. This argument is validated by the total number of air travelers per country.

Regarding cultural values, both regression analyses showed that countries with high individualistic values and high intellectual autonomy were found to be significantly and consistently associated with high COVID-19 deaths, whereas countries with higher collectivist values were associated with fewer COVID-19 deaths, both in July and December 2020. High collectivism will increase the likelihood to comply with Covid-19 protective guidelines, while individuals with high individualistic and person-focused values might be less likely to comply [see also Wolf et al. (2020)]. Our findings validate what Elgar et al. (2020) propose that some dimensions of social capital, such as caring for the community, lead to fewer deaths. Other research found that higher prosocial tendencies were related to an acceptance of making sacrifices such as accepting a temporary economic lockdown (Howard, 2021). While reiterating that cultural values influence how communities react to and behave in this pandemic [see also Seale et al. (2020)], our findings indicate that people who care primarily for themselves and have less regard for the consequences of their actions on others behave in ways that are related to personal gain, convenience, and enjoyment, which may increase exposure and risk to the virus. When this happens, the spread of COVID-19 increases, which then ultimately leads to an increase in the number of deaths. However, when a society unites and cares for all other people in their community in solidarity, people behave in ways that consider the consequences for other people's health and safety. As a result, the spread of COVID-19 is mitigated, which then leads to lower death rates.

Additionally, high Power-Distance/low-Egalitarianism was significantly related to a higher number of COVID-19 related deaths. Countries that are more egalitarian had a higher number of COVID-19 deaths in July and December. According to this finding, when citizens regard each other as equals and *do not* regard hierarchical roles in society, they might be less willing to follow policies that mitigate the spread of COVID-19. Our findings show that it is exactly this social aspect of compliance that is important and unique in preventing the virus spread during a pandemic (Wolf et al., 2020) as well as adopting avoidance behaviors that are associated with trust in government/authorities (Seale et al., 2020).

There is a sense of responsibility in those who govern and those who are governed. Therefore, there is a system of preventive and precautionary efforts, as seen in Conyon et al. (2020) where more preventive health behaviors are present due to lockdown policies that are stricter, and where COVID-19 death rates are found to be lower. On the other hand, when societies do not accept hierarchies as a given, there is more regard for each other as equals. In such a case, citizens might question their leaders more and pay little or no heed to leaders' policies or efforts. Consequently, COVID-19 can spread faster, and the number of deaths unfortunately increases.

The findings of this study are based on data obtained from two different value studies, Hofstede's study with over 116,000 respondents from 76 countries and Schwartz's study with over 67,000 participants from 75 countries, in addition to data on current COVID-19 cases and deaths. Although some of the data have been conducted decades ago, the cultural value dimensions have been replicated and validated many times (e.g., Van Nimwegen, 2002; Cheng et al., 2013). Moreover, even though research has shown an overall tendency for countries to be moving slightly toward individualism, cultural differences have remained quite stable over time (Manfredo et al., 2017; Santos et al., 2017). This is because the manner in which values are formed and sustained makes them fairly resistant to change. Further, although the study is correlational and no causation can be assumed, findings show significant relationships between cultural values worldwide and COVID-19 deaths.

Based on these findings, political leaders, organizations, communities, and families could stress the importance of the community aspect within society, interconnectedness, and collectivist caring in mitigating the pandemic. It is not primarily economic variables that can prevent COVID-19 deaths. It is not solely individual health-related factors, the primary of which is the BMI, that predict COVID-19 related deaths. It is noteworthy that, according to our findings, especially when pertinent variables are controlled for, it is the bottom-up cultural values in a country that are related to COVID-19 deaths. These values, which have remained quite stable over time (Manfredo et al., 2017; Santos et al., 2017), are significantly associated with mortality from COVID-19. In countries where individualism is predominant, there is a challenge to change what is believed and regarded as important. With COVID-19 as a precursor to change, countries would make significant strides against the increasing number of deaths when public health policies stress the common good, collective health, and valuing the community and each other. Change happens on the individual level—valuing individuals as separate and disconnected from other individuals cannot sustain the world in this nor any pandemic.

## Data Availability Statement

The original contributions presented in the study are included in the article, further inquiries can be directed to the corresponding author.

## Author Contributions

All authors listed have made a substantial, direct and intellectual contribution to the work, and approved it for publication.

## Conflict of Interest

The authors declare that the research was conducted in the absence of any commercial or financial relationships that could be construed as a potential conflict of interest.
